# Symptoms and antecedents of autism in children born extremely premature: a national population-based study

**DOI:** 10.1007/s00787-022-01953-4

**Published:** 2022-03-10

**Authors:** Silje Katrine Fevang Elgen, Madland Ada Røiseland, Elgen Irene Bircow, Maria Vollsæter, Mari Hysing

**Affiliations:** 1 Department of Pediatrics, Haukeland University Hospital, Bergen, Norway; 2 Department of Child and Adolescent Psychiatry, Haukeland University Hospital, Bergen, Norway; 3 Department of Clinical Science, Faculty of Medicine and Dentistry, University of Bergen, Bergen, Norway; 4 Department of Psychosocial Science, Faculty of Psychology, University of Bergen, Bergen, Norway; 5 Regional Centre for Child and Youth Mental Health and Child Welfare, NORCE Norwegian Research Centre, Bergen, Norway; 6 Department of Clinical Science, Section of Child and Adolescent Psychiatry and Pediatrics, University of Bergen, N-5021 Bergen, Norway

**Keywords:** Extremely preterm, Extremely low birth weight, Autism, Mental health, Antecedents

## Abstract

The objective of this study is to investigate the prevalence of autism (ASD) symptoms, i.e. , social difficulties, repetitive behaviors, and communicational problems, among children born extremely preterm (EP) compared to a reference group, and to investigate possible antecedents of ASD symptoms among EP children. Method is a national Norwegian cohort of 11 year old EP children, excluding those with intellectual disabilities, non-ambulatory cerebral palsy, blindness, and/or deafness. Parents and teachers reported ASD symptoms using The Autism Spectrum Screening Questionnaire (ASSQ). Social difficulties, repetitive behaviors, communicational problems, and a total ASSQ score were presented. Combined ratings on the ASSQ was defined as parent and/or teacher scoring the child ≥ 98th percentile of the reference group, which was the population-based Bergen Child Study. Of eligible children, 216 (64%) EP and 1882 (61%) reference children participated. EP children had significantly higher mean scores and combined ratings on social difficulties (14.5% vs. 4.1%, OR: 3.2), repetitive behaviors (23.7% vs. 4.0%, OR: 6.4), communicational problems (23.1% vs. 4.8%, OR: 5.4), and the total ASSQ score (18.3% vs. 3.4%, OR: 5.7) compared to reference children. Only no prenatal steroids, IQ 70–84, and mental health problems at 5 years of age were significantly associated with ASD symptoms at 11 years of age. EP children were at increased risk of social difficulties, repetitive behaviors, and communicational problems, and approximately one out of five were reported as high scorers of ASD symptoms. No prenatal steroids use, IQ in the lower range, and mental health problems at 5 years of age were associated with ASD symptoms.

**Supplementary Information:**

The online version contains supplementary material available at 10.1007/s00787-022-01953-4.

## Introduction

Social difficulties, repetitive behaviors, and communication deficits are the three main symptom domains of Autism Spectrum Disorder (ASD) [1]. The prevalence of ASD among children born extremely preterm (EP), defined as gestational age < 28 weeks or birth weight < 1000 g, has been estimated to 7–8% [2, 3], which is significantly higher than the expected prevalence rate of 0.7% in the general population [4]. EP born children without a diagnosis of ASD also have a higher load of ASD symptoms compared to term born children [5]. Children suffering from severe neurodevelopmental disabilities (NDD), i.e., non-ambulatory cerebral palsy (CP), intellectual disabilities (ID), blindness, and deafness, are at increased risk of ASD [6, 7]. EP children are at increased risk of severe NDD [8]. Interestingly, even after excluding those with severe NDD [9] and IQ < 85 [10], EP children are still at risk of ASD symptoms compared to controls.

Most studies investigating ASD symptoms in EP children report this as a total score [2, 3, 10–13]. However, one study of EP children [5] excluding those with ASD and IQ < 85 investigated the three main symptom domains of ASD separately, finding EP children at increased risk in all the domains compared to norm data. Another study including EP children regardless of cognitive function and ASD had similar findings [14]. However, after adjusting for IQ, repetitive behavior was no longer significantly different compared to term controls. Children with visual and hearing impairments and non-ambulatory CP were included in all the analyses in these two studies, bringing uncertainties to the findings [13]. To understand more fully what the reported ASD symptoms in EP children represents, further studies are needed.

The few studies investigating antecedents of ASD symptoms in EP children report bronchopulmonary dysplasia (BPD) [10, 15], jaundice [16], lower gestational age [14, 17], and neonatal brain abnormalities [14, 16, 17] to be associated with ASD symptoms in EP children. In addition, lower socioeconomic status (SES) [16], cognitive impairment, special educational needs, and poorer academic attainment [14] have been found to co-occur with ASD symptoms in EP children. Due to the increased risk of ASD symptoms in EP born, understanding the mechanisms and identifying possible antecedents would be valuable. 

The aims of the present study of a national cohort of EP children at 11 years of age were: (1) To investigate the prevalence and nature of symptoms and the three main symptom domains of ASD, among children born EP without non-ambulatory CP, intellectual disabilities, deafness or blindness compared to a reference group. (2) To investigate possible antecedents of ASD symptoms among EP children including pre, neonatal and perinatal factors, mental health problems, and functional outcomes at 5 years of age.

## Method

### Populations

#### EP group

The target population was a national cohort of all children born EP (gestational age (GA) < 28 weeks or birth weight (BW) < 1000 g) in Norway in 1999 and 2000 (*n* = 638). Of the 638 children born EP, 2 refused to participate, and 263 children died before 2 years of age. One child died between the 2 and 5 year follow-up. Both at 5 and 11 years of age, 372 (58% of the total) EP children were alive. At 5 years of age, 306 EP children attended somatic examination, and 255 of them completed mental health questionnaires. The EP children were prospectively assessed during their stay in the neonatal intensive care unit (NICU) and at 2, 5, and 11 years of age. At 2 and 5 years, they were assessed by experienced pediatricians, and at 5 years, psychologists assessed IQ using the Wechsler Preschool and Primary Scale of Intelligence-Revised (WPPSI-R) [18]. At 11 years of age, information was based on parent- and teacher-reported questionnaires. Characteristics of the cohort, definitions of neonatal characteristics, and overall outcome in terms of mortality and morbidity until 11 years of age have been published [19–25].

At 11 years of age, EP children were excluded if they at 5 years had an IQ < 70, non-ambulatory CP (class 4–5 on the Gross Motor Function for Classification of CP-GMFCS [26]), deafness, and/or blindness (*n* = 33) [22]. For children who did not participate at 5 but at 11 years of age (*n* = 35) [11], they were excluded based on information obtained from the 2 year follow-up [23]. None of the 35 children had CP, and were blind or deaf. An experienced pediatrician contacted the parents of 17 children by phone to obtain information on ID diagnosis, given that the information in the questionnaires were not sufficient. None of them had parent-reported ID diagnosis. One family was not approached, because the parents had not consented to further contact. A total of 338 EP children were eligible at 11 years of age, and of those 216 (64%) attended. Of them 161 (75%) were born with GA < 28, and 179 (83%) had BW < 1000 g. Furthermore, 69 of the 216 EP children were SGA, and of them, 31 (45%) children had mothers with preeclampsia. Parent-reported questionnaire was available for all participants, and the teacher report for 186 children. Regarding the assessed EP children, they had higher educated mothers, more BPD, less retinopathy, fewer were boys, and fewer had NDD and ambulatory CP at 5 years, compared to those not assessed [11].

#### Reference group

The reference group was the Bergen Child Study, an unselected population of 11-year-old children living in the municipality of Bergen, Norway [27, 28]. All children born in 1995 who attended public or private schools were invited, and 1893 (61%) participated. Children with ID, according to parent report (*n* = 11), were excluded. Six children were reported with CP without ID, and were included in the study, with unknown level of ambulation. Children with blindness or deafness without ID may have been included, as necessary information was lacking. A total of 1882 (61%) children were eligible and included in the analysis, including parents’ completed questionnaires for 1767 (57%) and teachers’ questionnaires for 1880 (61%) [11].

Comparing the attending children in the reference and the EP group, the number of fathers with higher education was significantly higher in the reference group, and father’s education was adjusted for in all the analyses comparing the two groups. Higher education was defined as at least 3 years of education at university level. There was no statistical significant difference between the proportion of mother’s with higher education and proportion of boys (see Online Resource 1) [11].

#### The autism spectrum screening questionnaire (ASSQ)

The Autism Spectrum Screening Questionnaire (ASSQ) is a 27-item questionnaire reflecting symptoms of ASD; social interaction problems, motor clumsiness, repetitive behavior, and tics [29–32]. The ASSQ was developed by Ehlers and Gillberg as a screening tool intended to identify children who need more comprehensive evaluation for suspected ASD. The test–retest stability for the ASSQ have previously been 0.94 for teachers and 0.96 for parents during a 2 week period [33]. The items can be allocated into three subscales as recommended by Ryland et al. [32]. The subscales are (1) social interaction difficulties; addressing challenges with friendship, prosocial behavior, and social communication (11 items); (2) repetitive behavior (tics/motor/OCD); repetitive, stereotype autism-associated symptoms, such as motoric problems and tics (8 items); (3) autistic style/ communication difficulties; addressing a social–cognitive style and language/communication difficulties (7 items). In addition, a Total Score were presented, including all the items. The 27 items are answered on a scale from “not true” (0), “sometimes true” (1), to “certainly true” (2), giving a possible range of scores from 0 to 54 in total. Higher scores indicate higher frequency of symptoms. One of the items (number 9) was by accident absent in the reference group, and therefore not included in our data. We substituted missing items with the mean individual score for all the items in ASSQ. No child missed more than one item. Both mean scores and cut-off scores ≥ 98th percentile of the reference group were presented. Due to the stepwise change in ASSQ scores, the exact 98 percentiles could not be found in all cases, and some of the scores were approximate. Parents and teachers cut-off score ≥ 98th percentile were > 8 for social interaction difficulties, > 3 and > 2 for repetitive behavior, > 6.7 and > 3 for autistic style, and > 16 and > 13 for the Total score, respectively. In addition, a high combined score for each scale is defined as parent and/or teacher score of the child ≥ 98th percentile for the reference group. A high combined total ASSQ score (when parent and/or teacher score the child ≥ 98th percentile of the reference group on the total score) has been shown to give the highest specificity (0.86) and sensitivity (0.91) for detecting ASD in a Norwegian study [30]. Where parent or teacher report was missing, the child was excluded from this analysis. The ASSQ has not been validated for children with severe CP, blindness, deafness, or IQ < 50.

### Statistical analyses

First, mean ASSQ scores with *t* tests, ASSQ scores ≥ 98 percentiles for the reference group, and the combined ASSQ score with *χ*^2^ tests were compared between the EP and the reference group (Table 1). All the analyses in Table 1 were adjusted for fathers’ higher education, performing regression analyses, logistic or linear as appropriate, with ASSQ outcome as the dependent variable, and group and fathers’ higher education as independent variables. Second, the proportion of all the single ASSQ items scored as 1 or 2 were analyzed in both groups with Chi-square tests. Furthermore, binary correlation analyses were performed separately for the EP and reference children between single items and the total ASSQ score ≥ 98 percentiles for both parents and teachers report. Finally, logistic regression analyses were performed individually for each possible antecedent factor for the EP children, with a combined total ASSQ score ≥ 98 percentiles for the reference group as dependent variable, to investigate potential antecedents separately. Not all the 186 children with a combined total ASSQ score ≥ 98 percentiles had information regarding the different antecedent factors, and therefore, the number included in the analysis differs; see Table 3 for more information. In addition, a false discovery rate is calculated and presented for the *p* values in Table 3.

**Table 1:** Mean scores, high scorers^a^, and combined scores^b^ on the Autism Spectrum Screening Questionnaire (ASSQ)^c^ for extremely preterm^d^ (EP) children compared to a reference group^e^ at 11 years of age

	EP	Reference	*P* value		
	Mean(SD)	Mean(SD)			
	*n*(Parents) = 216	*n*(Parents) = 1767			
	*n*(Teachers) = 186	*n*(Teachers) = 1880			
	*n*(Combined) = 186	*n*(Combined) = 1743			
Social difficulties					
Parent	2.1 (3.1)	1.2 (2.3)	< 0.001		
Teacher	2.7 (3.6)	0.9 (2.2)	< 0.001		
Repetitive behavior					
Parent	0.8 (1.5)	0.3 (1.0)	< 0.001		
Teacher	1.2 (2.1)	0.2 (0.9)	< 0.001		
Communication difficulties					
Parent	2.2 (2.3)	1.5 (1.8)	< 0.001		
Teacher	1.9 (2.2)	0.5 (1.1)	< 0.001		
Total score^f^					
Parent	5.0 (5.8)	3.0 (4.2)	< 0.001		
Teacher	5.8 (6.7)	1.7 (3.5)	< 0.001		
High and combined scores	EP %(n)	Reference %(n)	Exp(B)	95%CI	*P* value
Social difficulties					
Parents	7.4 (16)	2.4 (42)	3.0	1.6–5.8	0.001
Teachers	8.0 (15)	2.5 (46)	3.5	1.8–6.7	< 0.001
Combined	14.5 (27)	4.1 (71)	3.2	1.9–5.6	< 0.001
Repetitive behavior					
Parents	6.0 (13)	2.2 (38)	2.6	1.3–5.4	0.009
Teachers	19.8 (37)	2.5 (47)	10.7	6.5–17.7	< 0.001
Combined	23.7 (44)	4.0 (69)	6.4	3.8–10.7	< 0.001
Communication difficulties					
Parents	4.7 (10)	2.5 (44)	1.7	0.7–3.6	0.199
Teachers	20.3 (38)	3.0 (57)	7.5	4.7–12.2	< 0.001
Combined	23.1 (43)	4.8 (83)	5.4	3.5–8.4	< 0.001
Total Score^g^					
Combined	18.3 (34)	3.4 (60)	5.7	3.4–9.4	< 0.001

^a^Scores ≥ 98th percentile for the reference group

^b^Parent and/or teacher scoring the child ≥ 98th percentile for the reference group

^c^ASSQ is a questionnaire screening for ASD

^d^Gestational age 22–27 weeks or birth weight < 1000 g

^e^Children from the longitudinal population-based Bergen Child Study, born in 1995

^f^Total Score; a sum score of all ASSQ items. All analysis were adjusted for father’s education

^g^High scores reported by parents and teachers have previous been published (Fevang et al. 2016). Excluding the EP children with GA ≥ 28 did not change the results significantly (see Online Resource 2 for more information)

A *p* value of < 0.05 was considered statistically significant. The SPSS statistical package version 25.0 (IBM SPSS Statistics, IBM Corporation) was used for all analyses.

The Regional Committee on Medical Research Ethics and The Norwegian Data Inspectorate approved the study, and parents gave written informed consent.

## Results

### Total score of autism symptoms

EP children had significantly higher (*p* < 0.001) mean scores on both parent- and teacher-reported total ASSQ score and significantly increased odds of a high combined total ASSQ score (OR: 5.7; 95% CI 3.4–9.4) compared to the reference group (Table 1).

### Social interaction difficulties

Regarding social interaction problems reported by parents and teachers, the EP children had significantly higher (*p* ≤ 0.001) mean scores, increased odds of being scored ≥ the 98th percentile (OR: 3.0 and OR: 3.5, respectively), as well as odds (OR: 3.2) of having a combined social interaction problem score compared to the reference group (Table 1). Both parents and teachers reported “No social fit in language” (28.8 and 29.4%, respectively) as the most frequent individual item in the social interaction domain for the EP children. “Lacks common sense” was the single item with the highest correlation (0.56) to parent-reported total ASSQ score ≥ 98th percentile. “Poor at games, own rules” were the single item for the EP children with the highest correlation (0.66) to teachers reported total ASSQ score ≥ 98th percentile (Table 2).

**Table 2:** Proportions of extremely preterm^a^ (EP) and reference^b^ children reported with difficulties^c^ on an item from the Autism Spectrum Screening Questionnaire^d^ (ASSQ) reported by parents and teachers, and correlation between single items and the Total Score ≥ 98th percentile^e^ for the different groups

Items	Parents				Teachers			
	EP (%)	Reference (%)	EP correlation	Reference correlation	EP (%)	Reference (%)	EP correlation	Reference correlation
Repetitive behavior								
Different voice/speech	9.3	4.2**	0.31	0.28	22.5	4.4***	0.47	0.39
Clumsy	16.3	4.4***	0.38	0.42	30.5	5.7***	0.55	0.38
Involuntary movements	7	3.7*	0.39	0.29	7.5	1.8***	0.36	0.26
Compulsory repetitive	5.6	1.3***	0.3	0.5	3.2	0.5***	0.3	0.35
Insists on no change	9.8	4.0***	0.37	0.37	5.9	1.3***	0.37	0.28
Idiosyncratic attachment	13	6.5**	0.54	0.43	12.3	2.1***	0.38	0.40
Unusual facial expression	1.4	1.5	0.14	0.51	8	1.2***	0.41	0.32
Unusual posture	3.7	1.5*	0.32	0.47	8	1.5***	0.46	0.32
Social difficulties								
Lives in own world	19.1	11.5**	0.48	0.42	25.7	7.3***	0.58	0.52
No social fit in language	28.8	16.8***	0.52	0.38	29.4	7.5***	0.6	0.53
Lacks empathy	11.2	6.6*	0.4	0.48	12.8	6.9**	0.48	0.37
Naive remarks	15.3	9.2**	0.42	0.32	18.7	5.5***	0.46	0.45
Deviant style of gaze	15.3	6.4***	0.32	0.36	23	6.1***	0.37	0.37
Fails to make friends	18.1	7.8***	0.52	0.44	25.1	8.8***	0.54	0.50
Sociable on own terms only	14.4	11.1	0.42	0.36	16.6	8.8***	0.53	0.48
Lacks best friend	20	13.2**	0.35	0.23	24.1	15.2**	0.38	0.34
Lacks common sense	11.2	4.1***	0.56	0.46	11.8	4.1***	0.54	0.50
Poor at games, own rules	15.8	7.7***	0.34	0.43	24.1	9.3***	0.66	0.47
Bullied by other children	12.6	9.7	0.35	0.21	9.1	5.5*	0.4	0.32
Communication difficulties								
Old-fashioned or precocious	41.9	47.4	0.16	0.12	36.4	19.1***	0.17	0.12
Uneven abilities	36.7	23.3***	0.38	0.28	29.9	6.5***	0.55	0.46
Literal understanding	24.7	13.0***	0.42	0.33	23	4.3***	0.41	0.28
Accumulate facts	30.2	16.8***	0.38	0.3	26.7	4.5***	0.31	0.36
Eccentric professor	18.1	11.3**	0.25	0.21	17.6	5.4***	0.21	0.12
Robotlike language	7.9	3.5**	0.35	0.35	12.3	2.2***	0.46	0.30
Idiosyncratic words	10.2	14.7	0.27	0.12	7.5	1.2***	0.42	0.31

^a^Gestational age 22–27 weeks or birth weight < 1000 g

^b^Children from the longitudinal population-based Bergen Child Study, born in 1995

^c^scored with the value “sometimes true”(1) or “certainly true”(2)

^d^ASSQ is a questionnaire screening for ASD

^e^Total Score, including all the items on the ASSQ, scored ≥ 98th percentile for the reference group

***p value < 0.001, **p value < 0.01, *p value < 0.05

### Repetitive behavior (tics/motor/OCD)

For repetitive behavior reported by parents and teachers, the EP children had significantly higher mean scores (*p* < 0.001), odds of being scored ≥ the 98th percentile (OR: 2.6 and OR: 10.7, respectively), and odds (OR 6.4) of having a combined repetitive behavior score compared to the reference group (Table 1). “Clumsy” was the most frequent individual item reported by both parents and teachers (16.3 and 30.5%, respectively) (Table 2). “Idiosyncratic attachment” was the single item with the highest correlation (0.54) to parent-reported total ASSQ score ≥ 98th percentile, whereas “Clumsy” was the single item for the EP children with the highest correlation (0.55) to teachers reported total ASSQ score ≥ 98th percentile (Table 2).

### Autistic style/communication difficulties

Concerning autistic style, EP children had significantly higher (*p* < 0.001) mean scores reported both by parents and teachers, and increased odds (OR: 7.5) of being scored ≥ the 98th percentile reported by teachers, and increased odds (OR: 5.4) of having a combined autistic style score compared to the reference group. Parental report score ≥ the 98th percentile between the two groups was not significantly different (Table 1). Both parents and teachers reported “Old-fashioned or precocious” as the most frequent individual item (41.9 and 36.4%, respectively). “Literal understanding” was the single item with the highest correlation (0.42) to parent-reported total ASSQ score ≥ 98th percentile, and “Uneven abilities” was the single item for the EP children with the highest correlation (0.55) to teachers reported total ASSQ score ≥ 98th percentile (Table 2).

### Antecedents of a combined total score for the EP children

Of the 27 variables investigated as antecedents of a combined Total Score for the EP children, three were significantly associated with increased risk; no prenatal steroids (*p* = 0.002), IQ in the lower normal range (IQ 70–84) (*p* = 0.008), and mental health problems at 5 years of age (*p* = 0.001). After performing false discovery rate analysis, IQ in the lower normal range (IQ 70–84) (*p* = 0.008) was no longer significant (*p* = 0.072, Table 3).

**Table 3:** Investigating univariate factors from the pre-, peri, and neonatal period, and from 5 years of age, and the association with a Combined Total Score^a^ on the Autism Spectrum Screening Questionnaire^b^ (ASSQ) at 11 years of age for a national cohort of extremely preterm (EP)^c^ children born in Norway in 1999–2000

	Exp(B)	95% CI	*P* value	FDR*P value
Boys (*n* = 93/186)	1.3	0.6–2.8	0.449	0.673
Low maternal education at birth^d^ (*n* = 69/167)	0.7	0.3–1.5	0.376	0.597
Maternal age at birth (per year) (*n* = 186)	0.9	0.6–1.4	0.746	0.876
Birthweight (per 100 g) (*n* = 186)	0.9	0.7–1.2	0.538	0.755
GA (per week) (*n* = 186)	1.2	0.9–1.5	0.224	0.432
GA < 28 weeks (*n* = 145/186)	2.1	1.0–4.8	0.062	0.209
Small for GA, < 5 percentile^e^ (*n* = 34/186)	0.9	0.4–2.5	0.916	0.989
Prenatal steroids (*n* = 134/186)	0.3	0.1–0.6	0.002	0.027
Postnatal steroids (*n* = 62/186)	0.8	0.4–1.8	0.592	0.755
Preeclampsia (*n* = 47//186)	1.8	0.8–4.1	0.137	0.351
Chorioamnionitt (*n* = 21/186)	0.7	0.2–2.6	0.615	0.754
PROM^f^ (*n* = 23/176)	0.2	0.0 to 1.4	0.058	0.209
Surfactant (*n* = 33/186)	1.0	0.4–2.7	0.987	0.993
Multiple births^g^ (*n* = 47/186)	0.5	0.2–1.2	0.117	0.351
Bronchopulmonary dysplasia^h^ (*n* = 89/186)	1.7	0.8–3.7	0.156	0.351
NEC^i^ (proven or suspected) (*n* = 10/186)	1.1	0.2–5.6	0.885	0.989
No pathologic cerebral ultrasound^j^ (*n* = 126/186)	1.0	0.2–4.8	0.993	0.993
Retinopathy of prematurity (n = 45/186)	0.6	0.2–1.6	0.324	0.547
Peer problems (*n* = 44/141)	3.0	1.2–7.2	0.013	0.088
Emotional problems (*n* = 56/141)	2.7	1.1–6.7	0.022	0.119
Conduct problems (*n* = 40/141)	1.9	0.8–4.7	0.155	0.351
Hyperactivity/inattention (*n* = 70/141)	2.5	1.1–6.3	0.043	0.194
Total problems (*n* = 49/141)	4.5	1.8–11.1	0.001	0.043
Visual impairments^l^ (*n* = 34/158)	1.8	0.7 to 4.4	0.172	0.357
Hearing impairments^m^ (n = 23/158)	1.4	0.5 to 4.0	0.585	0.075
Motor problems^n^ (*n* = 34/152)	1.6	1.4 to 4.1	0.318	0.547
IQ 70–84^o^ (*n* = 29/129)	3.3	1.3 to 8.5	0.008	0.072

^a^Parent and/or teacher score the child ≥ 98th percentile from the reference group

^b^The Autism Spectrum Screening Questionnaire

^c^Gestational age (GA) 22–27 weeks or birthweight 500–999 g

^d^Less than 3 year college education or a university degree

^e^Birth weight < 5th percentile for gestational age

^f^Premature rupture of membrane

^g^Twins or more

^h^Assisted ventilation or oxygen supplementation at 36 weeks’ postconceptional age

^i^Necrotizing enterocolitis

^j^No periventricular hemorrhage or periventricular cysts

^k^from the Strengths and Difficulties Questionnaire with a score ≥ the 90^th^ percentile from the reference group, which were children born in 2001 attending the scheduled routine public health care program at 5–6 years of age in Oppland County, Norway

^l^but not blind

^m^hearing loss with/without need of hearing aid, but not deafness

^n^Movement Assessment Battery for children; score ≥ 95^th^ percentile, and not having cerebral palsy

^o^The Wechsler Preschool and Primary Scale of Intelligence-Revised. There is a possibility of type 2 error due to small cell counts. Regression analyses, logistic or linear were performed as appropriate, with combined total ASSQ score as dependent variable, and each of the possible antecedent variables separately as independent variables

*FRD false discovery rate *p* value

## Discussion

The present study found children born EP without non-ambulatory CP, intellectual disabilities, deafness, or blindness to be at a twofold to more than tenfold increased risk of exhibiting autism symptoms compared to reference children. Almost one in five EP children were rated with a combined Total ASSQ score ≥ 98th percentile. Repetitive behavior symptoms were reported with the highest risk of the three ASD symptom domains compared to the reference children. The single ASSQ item with the highest correlation to the Total ASSQ score ≥ 98th percentile for parents was “Lacks common sense” and for teachers “Poor at games, own rules”. Antecedents for ASD symptoms at 11 years of age were not receiving steroids prenatal, IQ in the lower normal range, and mental health problems at 5 years of age.

Strengths of the present study were the large national population-based sample of EP children followed up repeatedly until 11 years of age, the use of a large unselected reference group, multi-informants for autism symptoms, the prospective design, the examination at 5 years of age by psychologist, pediatrician and physiotherapist, substantial information from the NICU, and a small variation in age at assessment. Another strength of the study was the exclusion of children with severe NDD, thereby reducing the proportion of false positives [13]. However, this has possibly excluded most children with ASD from the EP sample, as ID and severe CP often co-occur in EP children with ASD [10]. As Korzeniewski et al. [5], the present study may therefore mainly describe EP children without ASD and ID affecting the outcome of the different main areas of ASD symptoms. A weakness was the use of a screening tool, and not a diagnostic tool, for autism. Further weaknesses were suboptimal follow-up rates, which also limited the power to adjust for additional potentially confounding factors, and lack of data on the eligible reference children who did not participate. However, at 7–9 years of age, it was estimated that the reference participants had fewer mental health symptoms than the nonparticipants [34]. This may also be the case for the EP group, since the attending children had a higher proportion of mothers with higher education, which is associated with less-frequent mental health problems for their children [19]. In addition, some children with ID may have been included both in the EP and the control group, since IQ was not tested at 11 years. Finally, we cannot exclude that the loss of 1 item on the ASSQ may have reduced the validity of the findings.

In accordance with one EP study excluding those with ASD and IQ < 85, the present study found increased risk of all the main symptom domains of ASD for the children born EP compared to the reference group [5]. This is in contrast to another EP study, where Johnson et al. found increased risk of social difficulties and communication deficits, and not repetitive behavior after adjusting for IQ [14]. Interestingly, in accordance with a study of very preterm born children (GA < 32) [35], the present study found repetitive behavior to be the ASD symptom domain with the highest risk for the EP children. However, the most frequent item in the repetitive symptom domain reported by both parents and teachers in the present study was being “Clumsy”. Children born EP are at increased risk of minor motor problems and clumsiness [36], and the high rate of repetitive behavior may be due to clumsiness and not due to ASD. This may reflect a challenge in EP research due to the high prevalence of comorbid conditions with overlapping symptoms. Furthermore, a contributing factor to the differing outcome to Johnson et al., may be their lack of teacher’s informants, as teachers in the present study reported a considerably greater symptom load of repetitive behavior than parents. Finally, the use of different screening tools in these studies may be a possible explanation for the differing outcomes.

To our knowledge, no study in EP children has previous investigated single symptoms of ASD. A study of adolescents born with very low birth weight (VLBW ≤ 1500 g) [37] found them to be high scorers on the ASSQ items; ‘‘Uses language freely, but fails to make adjustment to fit social contexts or the needs of different listeners’’ [37], which in the present study both parents and teachers reported as the most frequent individual item in the social interaction domain. Korzeniewski et al. investigated ASD symptoms in EP children without ASD [5], and found those with a high ASD score to more often have deficits in language and communication. This may suggest that communicational problems represent a great deal of the increased frequency of ASD symptoms previously reported. Furthermore, the VLBW study [37] reported them to be high scorers on the ASSQ items; “Fails to make friends” which was highly correlated to a total ASSQ score in the present study. There might be several explanations for failing to make friends. In addition to language and communication problems, EP children are at increased risk of inattention, emotional problems [11], and lower cognitive function [8], important domains affecting peer relationships. Detecting and helping EP children in need of support for socialization are important, as having friends is a fundamental part of quality of life including social activity and participation.

In accordance with one previous EP study [14], we found IQ in the lower normal range (70–84) and mental health problems at preschool age to be significant predictors of ASD symptoms at 11 years of age. This may not surprise as ASD occurs early in life and lasts a lifetime, and often co-exist with lower cognitive function and other mental health problems [38]. However, for those without ASD, this may reflect a cognitive and mental vulnerability early in childhood being a risk factor for later mental health problems, such as ASD symptoms or social difficulties. This could also be explained by the demands in social interaction, increasing with age and becoming more obvious for those surroundings the EP child.

In contrast to previous studies investigating neonatal factors [10, 14–16], the present study found that only not receiving prenatal steroids correlated with ASD symptoms at 11 years of age. The contradicting findings may be due to differences in statistical strategies. Interestingly, the previous findings reflects an additional vulnerability in the neonatal period which may affect the development of the brain causing ASD symptoms among other things.

## Conclusion

In this study of Norwegian children born EP without severe functional disabilities are at a twofold to tenfold increased risk of very high scores on all main symptom domains of ASD. Almost every fifth EP child had high scores of ASD symptoms. Not receiving prenatal steroids, IQ in the lower normal range, and mental health problems at 5 years of age were predictors of ASD symptoms at 11 years. However, limitations to the study should be taken into account regarding generalizability to other settings. Understanding the complex pattern of potential mental health problems is important for parents, health care personnel, teachers, and others who relate to children born EP.

## Supplementary Information

Below is the link to the electronic supplementary material.

Supplementary file1 (PDF 429 KB)

Supplementary file2 (PDF 511 KB)

## Data Availability

In accordance with the approvals granted for this study by The Regional Committee on Medical Research Ethics and The Norwegian Data Inspectorate, the data files are stored securely and in accordance with the Norwegian Law of Privacy Protection. The data file cannot be made publicly available as this might compromise the respondents’ privacy. Some of the participating centers are small and the number of extremely preterm births limited with a risk of identifying pseudonymous participants. To prepare future research papers, other researchers in our group currently use the data file. A subset of the data file with anonymized data can be made available to interested researchers upon reasonable request to Maria Vollsæter (maria.vollseter@helse-bergen.no), providing that Norwegian privacy legislation and GDPR are respected, and that permission is granted from The Norwegian Data Inspectorate and the data protection officer at Haukeland University Hospital.

## Abbreviations

EP: Extremely premature
ASD: Autism spectrum disorder
ID: Intellectual disabilities
CP: Cerebral palsy
NICU: Neonatal intensive care unit
ASSQ: The Autism Spectrum Screening Questionnaire
