# Correction to: Symptoms and antecedents of autism in children born extremely premature: a national population-based study

**DOI:** 10.1007/s00787-022-02026-2

**Published:** 2022-06-21

**Authors:** Silje Katrine Fevang Elgen, Madland Ada Røiseland, Elgen Irene Bircow, Maria Vollsæter, Mari Hysing

**Affiliations:** 1 Department of Pediatrics, Haukeland University Hospital, Bergen, Norway; 2 Department of Child and Adolescent Psychiatry, Haukeland University Hospital, Bergen, Norway; 3 Department of Clinical Science, Faculty of Medicine and Dentistry, University of Bergen, Bergen, Norway; 4 Department of Psychosocial Science, Faculty of Psychology, University of Bergen, Bergen, Norway; 5 Regional Centre for Child and Youth Mental Health and Child Welfare, NORCE Norwegian Research Centre, Bergen, Norway; 6 Department of Clinical Science, Section of Child and Adolescent Psychiatry and Pediatrics, University of Bergen, N-5021 Bergen, Norway

## Correction to: European Child & Adolescent Psychiatry 10.1007/s00787-022-01953-4

The original article has published with incorrect first, fourth and fifth author names. The correct names are Silje Katrine Fevang Elgen, Maria Vollsæter, Mari Hysing.

The original article has been corrected.

