# Supplementary material for: Symptoms and antecedents of autism in children born extremely premature: a national population-based study
Source: Eur Child Adolesc Psychiatry. 2022 Mar 10;32(9):1579–88. doi: 10.1007/s00787-022-01953-4 (PMC10460365; doi:10.1007/s00787-022-01953-4)
Supplement: Supplementary file 1 — Supplementary file1 (PDF 429 KB) [file 787_2022_1953_MOESM1_ESM.pdf]

Symptoms and antecedents of autism in children born extremely premature

A national population based study

Journal: European Child & Adolescent Psychiatry. Authors: Fevang Silje Katrine Elgen, Madland Ada Røiseland, Elgen Irene Bircow, Vollsæter Maria, Hysing Mari. Corresponding author: Silje Katrine Elgen Fevang, Department of Clinical Science, Section of Child and Adolescent Psychiatry and Pediatrics. University of Bergen, N-5021 Bergen, Norway. E-mail: silje.katrine.elgen.fevang@helse-bergen.no

Supplementary table 1: Comparison of the attending EP[a] and reference children[b] assessed at 11 years of age.

	EP children (n[c]) n (%)	Reference children (n[d]) n (%)	OR	95% CI	*p*-value[e]
Mother high education[f, g]	112 (59)	901 (54)	1.3	0.9-1.7	0.157
Father high education[f, g]	79 (42)	842 (52)	0.7	0.5-0.9	0.007
Boys	103 (49)	824 (47)	0.9	0.7-1.2	0.486

[a] Gestational age 22-27 weeks or birth weight <1000g, [b] Children from the longitudinal population-based Bergen Child Study in Norway, born in 1995, [c] EP: mother high education: n=189, father high education; n=190, boys; n= 209, [d] BIB: mother high education: n=1673, father high education: n=1621, boys; n=1764, [e] χ^2 test, [f] Education when the child was 11 years old, [g] At least 3 years of college education at university degree.
