# Supplementary material for: Symptoms and antecedents of autism in children born extremely premature: a national population-based study
Source: Eur Child Adolesc Psychiatry. 2022 Mar 10;32(9):1579–88. doi: 10.1007/s00787-022-01953-4 (PMC10460365; doi:10.1007/s00787-022-01953-4)
Supplement: Supplementary file 2 — Supplementary file2 (PDF 511 KB) [file 787_2022_1953_MOESM2_ESM.pdf]

Symptoms and antecedents of autism in children born extremely premature

A national population based study

Journal: European Child & Adolescent Psychiatry. Authors: Fevang Silje Katrine Elgen, Madland Ada Røiseland, Elgen Irene Bircow, Vollsæter Maria, Hysing Mari. Corresponding author: Silje Katrine Elgen Fevang, Department of Clinical Science, Section of Child and Adolescent Psychiatry and Pediatrics. University of Bergen, N-5021 Bergen, Norway. E-mail: silje.katrine.elgen.fevang@helse-bergen.no

Supplementary table 2: Mean scores, high scorers[a] and combined scores[b] on the Autism Spectrum Screening Questionnaire (ASSQ)[c] for extremely preterm[d] (EP) children (only included those with gestational age 22-27) compared to a reference group[e] at 11 years of age.

	**EP**<br>Mean(SD)	**Reference**<br>Mean(SD)	*p*-value
	N(Parents)=161<br>N(Teachers)=143<br>N(Combined)=143	N(Parents)=1767<br>N(Teachers)=1880<br>N(Combined)=1743	
**Social difficulties**			
Parent	2.1(3.1)	1.2(2.3)	<0.001
Teacher	2.5(3.4)	0.9(2.2)	<0.001
**Repetitive behavior**			
Parent	0.8(1.5)	0.3(1.0)	<0.001
Teacher	1.2(1.9)	0.2(0.9)	<0.001
**Communication difficulties**			
Parent	2.1(2.3)	1.5(1.8)	<0.001
Teacher	1.8(2.2)	0.5(1.1)	<0.001
**Total score[f]**			

Parent	5.0(5.8)	3.0(4.2)			<0.001
Teacher	5.5(6.3)	1.7(3.5)			<0.001
	**EP**	**Reference**			
High and combined scores	%(n)	%(n)	Exp(B)	95%CI	*p*-value
**Social difficulties**					
Parents	6.8(11)	2.4(42)	2.5	1.2-5.6	0.020
Teachers	7.0(10)	2.5(46)	3.2	1.5-6.9	0.002
Combined	12.6(18)	4.1(71)	2.9	1.6-5.4	0.001
**Repetitive behavior**					
Parents	6.2(10)	2.2(38)	2.5	1.1-5.9	0.029
Teachers	21(30)	2.5(47)	11.7	6.8-20	<0.001
Combined	24.5(35)	4.0(69)	7.5	4.6-12.2	<0.001
**Communication difficulties**					
Parents	5(8)	2.5(44)	1.7	0.7-4.2	0.221
Teachers	16.8(24)	3.0(57)	5.7	3.2-10.0	<0.001
Combined	19.6(28)	4.8(83)	4.2	2.5-7.0	<0.001
**Total Score**					
Parents	5.6(9)	2(35)	2.8	1.2-6.4	0.018
Teachers	12.6(18)	2(38)	7.1	3.7-13.6	<0.001
Combined	15.4(22)	3.4(60)	4.5	2.5-8.0	<0.001

[a]Scores ≥98th percentile for the reference group, [b]Parent and/or teacher scoring the child ≥98th percentile for the reference group, [c]ASSQ is a questionnaire screening for ASD, [d]Gestational age 22-27 weeks. [e]Children from the longitudinal population-based Bergen Child Study, born in 1995, [f]Total Score; including all the items on the ASSQ form. All analysis were adjusted for father's education.
